# Aerobic Exercise Training and VO_2max_: A Scoping Review of Study Populations and Protocols

**DOI:** 10.3390/jfmk11010070

**Published:** 2026-02-10

**Authors:** Zeyu Wu, Nicholas Preobrazenski, John R. M. Renwick, Ava Khansari, Matisse A. LeBouedec, Jared M. G. Nuttall, Ahmed Mudwi, Brendan Ross, Nia Simpson-Stairs, Lucas P. R. Beaupre, Paul A. Swinton, Brendon J. Gurd

**Affiliations:** 1 School of Kinesiology and Health Studies, Queen’s University, Kingston, ON K7L3N6, Canada; 18zw1@queensu.ca (Z.W.); 12np22@queensu.ca (N.P.); renwickj@mcmaster.ca (J.R.M.R.); 19ak127@queensu.ca (A.K.); matisse.lebouedec@queensu.ca (M.A.L.); 18jmgn@queensu.ca (J.M.G.N.); a.mudwi@queensu.ca (A.M.); brendan.ross@queensu.ca (B.R.); nsimp089@uottawa.ca (N.S.-S.); l.beaupre@queensu.ca (L.P.R.B.); 2 Faculty of Medicine, University of Ottawa, Ottawa, ON K1H 8M5, Canada; 3 School of Health, Robert Gordon University, Aberdeen AB10 7QE, UK; p.swinton@rgu.ac.uk

**Keywords:** aerobic training, VO_2max_, MICT, HIIT, SIT, sex-representation

## Abstract

Maximal aerobic capacity (VO_2max_) is a well-established predictor of cardiovascular health, morbidity, and all-cause mortality. While many systematic reviews and meta-analyses have characterized the effects of aerobic exercise training on VO_2max_, they fail to capture the state of the literature as a whole. This scoping review aims to summarize the populations and training protocols used in the current literature and highlight gaps in our current understanding of the VO_2max_ response to aerobic training. A total of 617 studies were selected and analyzed in this review. The majority of exercise protocols used were moderate intensity continuous training (MICT; n = 363). Few studies employed high-intensity interval training (HIIT; n = 102), sprint interval training (SIT; n = 70), or a combination of exercise modalities (n = 82). A large number of studies only included male participants (n = 264), while a few studies only included female participants (n = 83). The majority of training interventions were shorter than three months (n = 399). Many studies failed to report information regarding participant health (n = 169) and physical activity status (n = 290). Exercise modality, sex representation, the effects of long-term training, and reporting practices represent key gaps within the literature that should be further explored in the future.

## 1. Introduction

Maximal aerobic capacity (VO_2max_) has remained a central focus of exercise physiology research since its initial description in 1923 [1]. Decades of experimental work have explored both the mechanisms determining VO_2max_ [2] and its links to exercise performance [3]. VO_2max_ provides a quantitative measure of cardiorespiratory fitness (CRF), reflecting the integrated capacity of the cardiovascular, respiratory, and skeletal muscle systems to transport and utilize oxygen to support physical work. Recently, the American Heart Association released a statement highlighting CRF (and by proxy VO_2max_) as an important and underutilized vital sign [4]. Clinically, low VO_2max_ is recognized as an independent predictor of morbidity and mortality [5], and improvements in VO_2max_ following exercise interventions are associated with a decreased risk of all-cause mortality [6]. Exercise interventions aimed at improving VO_2max_ have traditionally emphasized low- to moderate-intensity continuous aerobic exercise [1], with more recent studies extending this focus to include higher-intensity, interval-based protocols [6]. Given the breadth of exercise approaches now employed to improve VO_2max_, the central question for clinicians and researchers is not whether VO_2max_ is important, but which aerobic training protocols increase VO_2max_ most effectively across different populations.

Given its physiological and clinical significance, the literature examining the impact of exercise training on VO_2max_ is unsurprisingly extensive. A PubMed search conducted on 7 January 2026 for ‘exercise training’ and ‘VO_2max_’ yielded 12,030 results. Numerous recent reviews have summarized aspects of this literature in various groups, including healthy adults [7], females [8], older adults [9], and individuals with Type 2 Diabetes [10]. Meta-analyses have also investigated the physiological mechanisms influencing VO_2max_, including capillarization [11] and central cardiovascular function [12]. However, existing reviews on VO_2max_ and exercise training have typically focused on specific subpopulations, individual mechanisms, or training variables in isolation without capturing the scope and breadth of the evidence base [13,14,15,16]. Despite the substantial diversity across exercise training studies, recurring patterns in the literature have not been systematically characterized, limiting the ability to contextualize where evidence is concentrated and where meaningful gaps remain. At present, a synthesis that formally maps the populations and training protocols examined in the VO_2max_ literature is lacking.

Therefore, as a first step in a larger project aimed at quantitatively synthesizing the effects of aerobic exercise training on VO_2max_, we conducted a scoping review to map the breadth, density, and underrepresented populations and training protocols within the existing literature. By clearly identifying research gaps and concentrations, this scoping review aims to inform future research priorities, support innovation, and reduce redundancy in addressing unanswered questions in improving VO_2max_.

## 2. Materials and Methods

This scoping review followed the Preferred Reporting Items for Systematic Reviews and Meta-Analyses (PRISMA-ScR) checklist (Supplementary Documents S1 and S2). The review selection process was conducted using Covidence’s systematic review software (Veritas Health Innovation, Melbourne, Australia). This review originated from a broader ongoing project; an a priori protocol was registered on Open Science Framework (osf.io/x9vu3). An initial publication from this larger project examined inter-individual differences in VO_2max_ trainability using a subset of studies included within this scoping review (n = 24) [17].

### 2.1. Eligibility Criteria

Studies were included in the scoping review if they met all of the following inclusion criteria: (1) experimental study (i.e., allocation to an aerobic training intervention), (2) enrolled human participants (≥18 years), (3) measured VO_2max_ (or VO_2peak_) using indirect calorimetry (i.e., cardiopulmonary exercise test), (4) reported absolute or relative VO_2max_ (if body weight was reported), (5) reported mean and standard deviation (SD) or standard error (SE) of VO_2max_ at PRE- and POST-training (or if these values could be calculated), (6) prescribed exercise intensity as a % of VO_2max_ or maximal work rate/power output (WR_max_; PO), and (7) used only supervised training protocols (i.e., direct, in-person oversight by study personnel). Studies were excluded if they: (1) did not meet all inclusion criteria, (2) prescribed a mixed training protocol (i.e., continuous + high-intensity interval training [HIIT]), (3) combined aerobic exercise with resistance training, (4) were not published in English, (5) were not original research, (6) were conference abstracts, or (7) were unavailable through institutional or publicly accessible databases. These eligibility criteria were selected to support a future meta-analysis by reducing heterogeneity related to participant age, exercise prescription, and outcome assessment.

### 2.2. Literature Search and Study Selection

The literature search was performed in EMBASE, PubMed, and SCOPUS using a search strategy combining terms related to “VO_2max_” and “exercise training”. A complete list of synonyms/related terms for our search strategy can be found in the Supplementary Document S3. The first search was performed on 2 July 2021, and the second on 7 February 2024, to identify more recent studies published since the initial search. Titles and abstracts were extracted from database searches and exported into Covidence, where duplicate records were automatically identified and removed.

Study selection followed a two-step process and was independently completed by eleven reviewers. First, titles and abstracts were screened to identify studies that appeared to meet eligibility criteria. Second, full texts were downloaded for articles that passed title and abstract screening to determine their eligibility. Studies removed during full-text screening were assigned a reason for exclusion. Final analyses included studies that passed both levels of study selection. Agreement between two independent reviewers was required at each step of the study selection process. Conflicting decisions were resolved by a third reviewer, or consensus was reached between the disagreeing reviewers.

**Figure 1:**
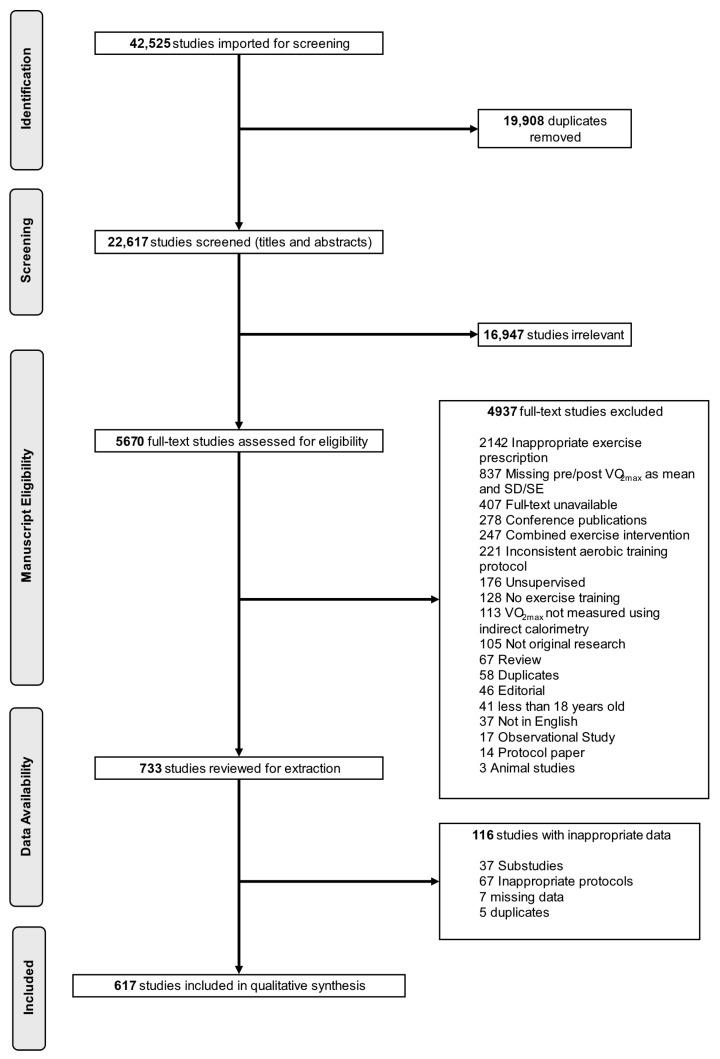
Flow diagram of study selection process. SD, standard deviation; SE, standard error; VO_2max_, maximal oxygen uptake.

A flow diagram of the study selection process is presented in Figure 1. Of the 22,617 studies that entered title and abstract screening (37,792 identified in July 2021 and 4733 in February 2024), only 5670 studies met the criteria for full-text screening. 617 studies of which met the inclusion criteria for this scoping review.

### 2.3. Data Extraction

Data extraction was performed using a predetermined data collection template on Covidence. A minimum of two independent reviewers was required to complete the extraction of any given study. Discrepancies between extracted data values were resolved by one reviewer. Study characteristics, including year of publication, country of origin, participant characteristics, sample sizes, training protocols, measured outcomes, and standard deviations (SD) of baseline and post-intervention values. A full list of extracted variables with descriptions is provided in Supplementary Document S4.

### 2.4. Risk of Bias Assessment

Consistent with the exploratory nature of scoping reviews, we did not conduct a formal risk of bias assessment for all included studies, as our primary aim was to map the breadth and characteristics of the literature rather than evaluate study quality. However, to provide some insight into methodological rigour, we applied the Cochrane Risk of Bias 2 (RoB 2) tool to a randomly selected ~10% subset of studies (n = 65) (see Supplementary Document S5). Two independent reviewers evaluated each study across five domains (e.g., randomization, outcome measurement, missing data) and categorized judgments as “low risk,” “some concerns,” or “high risk” [18]. Any disagreements between reviewers were resolved through discussion and consensus.

### 2.5. Data Analysis

All extracted data from Covidence were exported to an Excel spreadsheet (version 2506, Microsoft Corporation, Redmond, WA, USA). Macros created using Microsoft Visual Basic (version 16.9, Microsoft Corporation, Redmond, WA, USA) were used to format the data and identify additional extraction errors (Supplementary Document S6). GraphPad Prism (version 10.3.1, GraphPad Software, Boston, MA, USA) was used to depict data.

A citation analysis was also performed by the review team to map seminal works and describe patterns of publication and collaborations. Using the DOI extracted from each study and the metadata made available by Scopus (i.e., citation, bibliographical, and author information), the citation analysis was completed using R (version 4.5.1, R Core Team, Vienna, Austria).

## 3. Results

A total of 617 studies were included in this review. Supplementary Document S7 presents a summary of study characteristics.

### 3.1. Citation Analysis

A citation analysis was performed on the Scopus information obtained from 614 of the 617 total included studies. The citation analysis comprised journal of publication, citation counts, co-authorship networks, and keyword mapping (Supplementary Document S8). The studies were most frequently published in Medicine & Science in Sports & Exercise (n = 46, 7.5%), followed by the European Journal of Applied Physiology (n = 41, 6.7%), and then the Journal of Applied Physiology (n = 40, 6.5%). Belardinelli et al. [19] generated the most citations per year at 39, followed by Montero et al. [20] at 36, and Balducci et al. [21] at 31.

### 3.2. Country of Origin and Aerobic Training Modality

**Figure 2:**
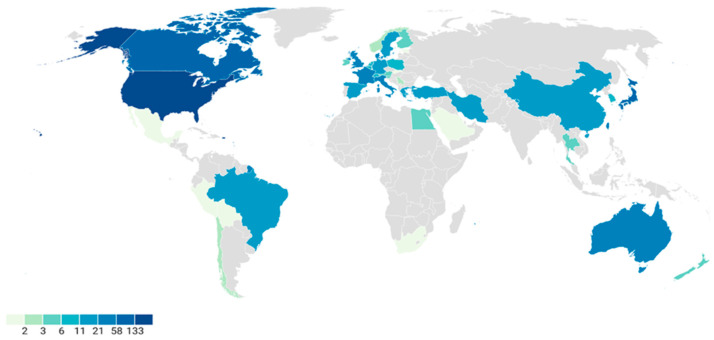
Distribution of publication frequencies (n = 617) across the world.

Supplementary Document S9 presents the frequency of studies published based on their country of origin. Studies were most often published by authors originating in the United States (n = 133, 21.5%), which was nearly double that of the next highest contributor, Canada (n = 73, 11.8%). Figure 2 presents a graphical representation of publication frequency by country of origin.

**Figure 3:**
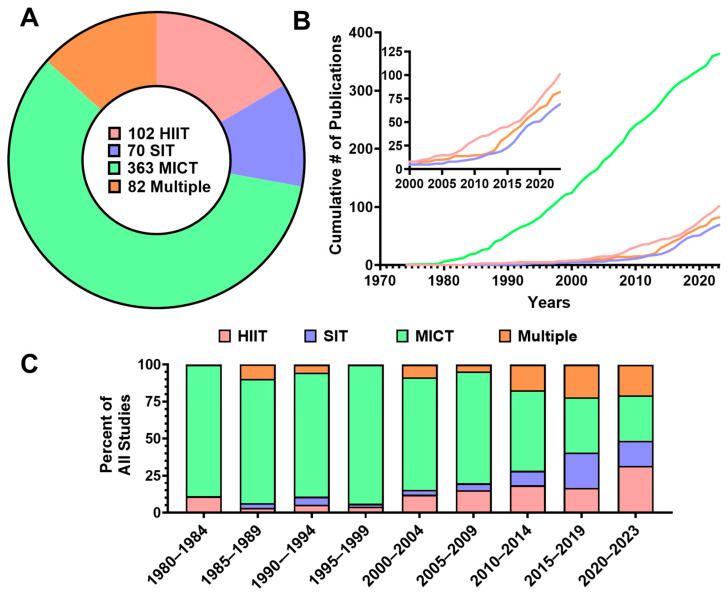
Trends in aerobic training modalities across VO_2max_ literature (n = 617). (**A**) Donut chart reveals overall frequency of each aerobic training modality included in the review: moderate-intensity continuous training (MICT), high-intensity interval training (HIIT), sprint interval training (SIT), and interventions incorporating multiple modalities. (**B**) Cumulative number of publications over time by aerobic training modality. (**C**) Temporal distribution of modalities expressed as a percentage of total studies within each 5-year interval.

Figure 3 presents frequency analyses of prescribed aerobic training modalities. Studies including moderate-intensity continuous training (MICT) were most prevalent (n = 363, 58.9%), with lower representation of HIIT (n = 102, 16.5%) and sprint interval training (SIT) (n = 70, 11.3%). Eighty-two studies (13.3%) included multiple aerobic training modalities (Figure 3A). Trends in the cumulative frequency of each aerobic exercise modality (from 1970 to 2023), and the percentage change in each aerobic exercise modality over time are presented in Figure 3B and Figure 3C, respectively.

### 3.3. Participant Sex

**Figure 4:**
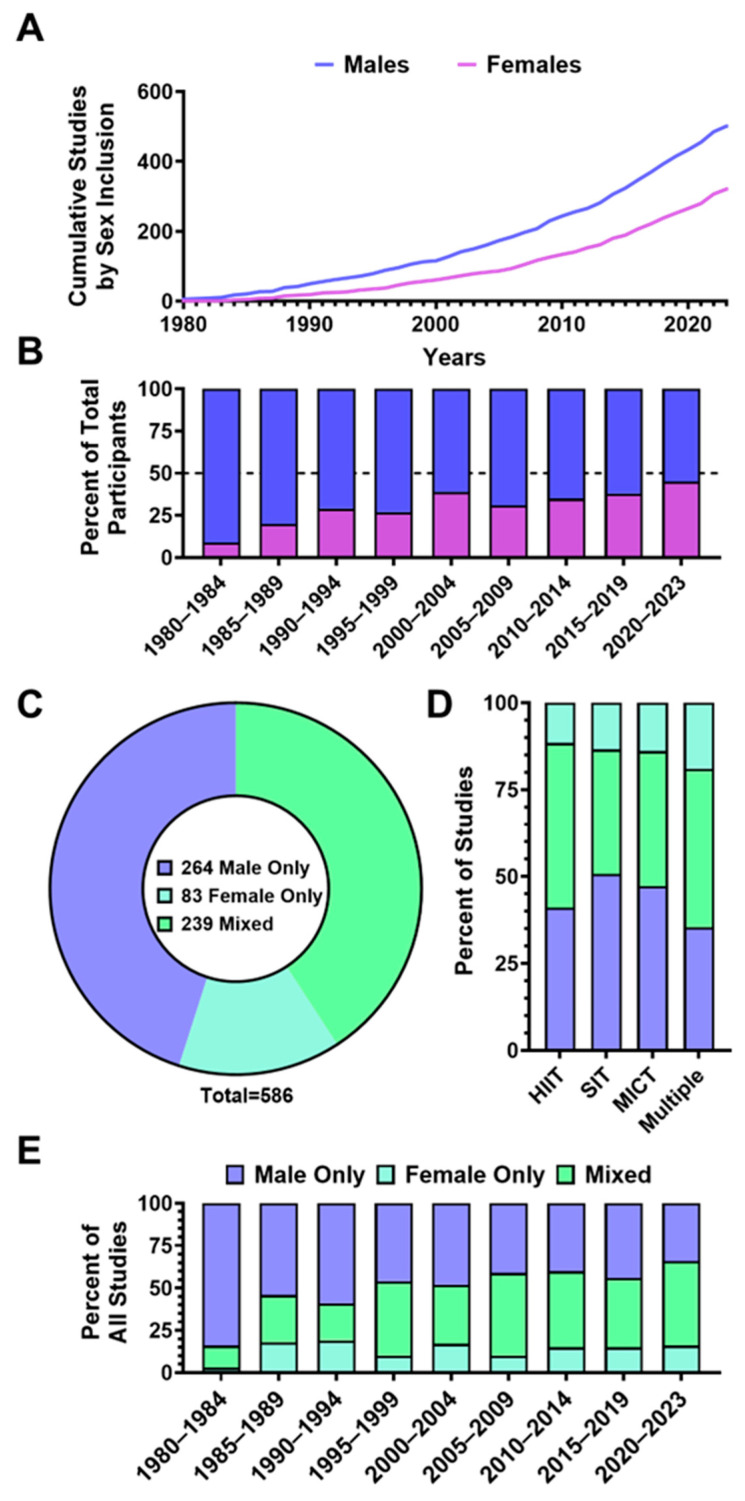
Trends in sex representation within aerobic training VO_2max_ literature (n = 586). (**A**) Cumulative number of studies including male (blue) and female (purple) participants over time. (**B**) Proportion of the total number of male and female participants across 5-year publication intervals. (**C**) Overall number of studies that included male-only, female-only, or mixed-sex cohorts. (**D**) Proportion of study types (male-only, female-only, mixed) by exercise modality. (**E**) Proportions of participant sex inclusion by study design across 5-year publication intervals.

Figure 4 depicts representation by sex in the included studies. Of the 617 included studies, 31 (5%) failed to report the sex of the participants. From the remaining studies (n = 586), a total of 17,893 participants were included, of which 11,617 were male (64.9%) and 6276 were female (35.1%). The number of studies that included males (n = 503, 85.8%) was approximately 50% greater than the number of studies that included females (n = 322, 54.9%) (Figure 4A). The number of males and females as a percentage of total participants per 5-year bin is presented in Figure 4B. Studies involving only males constituted a larger portion (n = 264, 45.1%) of the total number of studies compared with mixed sex (n = 239, 40.7%) and female-only (n = 83, 14.2%) (Figure 4C). Figure 4D shows the percent of male-only, female-only, and mixed studies across all years by aerobic exercise modalities, and Figure 4E depicts the changes in sex-based inclusion in all aerobic exercise modalities over time. Of the 239 mixed-sex studies, only 18 (7.5%) included an equal number of male and female participants. Most were male-biased (n = 141, 59.0%), whereas 80 studies (33.5%) included more female participants. Male-bias in participant ratio of mixed sex studies was evident across the included studies’ timeline.

### 3.4. Training Durations

**Figure 5:**
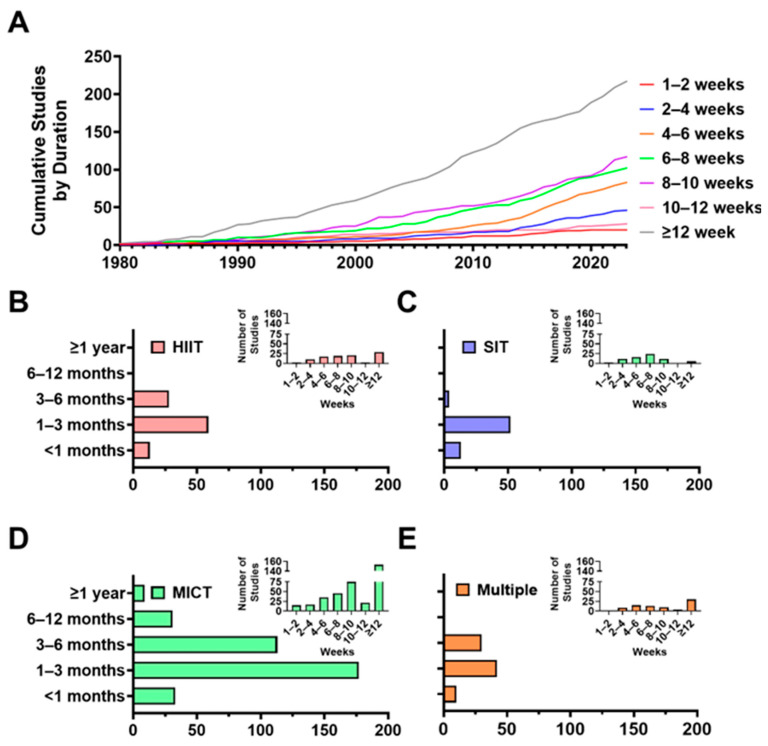
Trends in training durations across aerobic training VO_2max_ literature (n = 616). (**A**) Cumulative number of studies published over time, grouped by training durations in weeks. (**B**–**E**) Frequency of study durations across four exercise modalities (HIIT, SIT, MICT, Multiple).

Frequency analyses of training durations are presented in Figure 5. The cumulative number of studies, organized by training duration, is presented in Figure 5A. The most common training durations were ≥12 weeks (n = 217, 35.2%), followed by those that were 8–10 weeks (n = 117, 19%) and 6–8 weeks (n = 102, 16.6%). Across aerobic training modalities (Figure 5B–E), most studies prescribed training durations of 1–3 months (n = 330, 53.6%). However, the majority of HIIT (n = 72, 71.3%), SIT (n = 65, 92.9%), and multiple modalities (n = 52, 63.4%) studies were less than 12 weeks (as seen within insets). Thirty-one MICT studies employed training durations of 6–12 months, while only one HIIT and SIT study employed training durations of at least 6 months. Only MICT (n = 9) employed training durations ≥ 12 months.

### 3.5. Participant Age, Health, and Physical Activity Level

**Figure 6:**
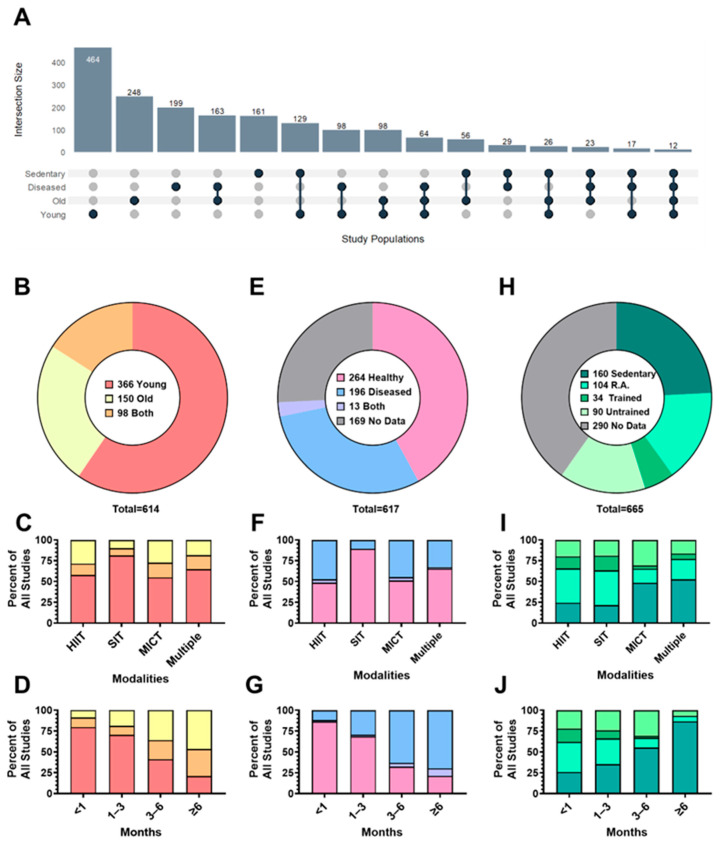
Trends in participant characteristics across aerobic training VO_2max_ literature (n = 617). (**A**) Upset plot illustrating the frequency and intersections of different participant characteristics (Young, Old, Diseased, Sedentary) included in studies examining VO_2max_ responses to exercise training. Each vertical bar represents the number of studies sharing a specific combination of participant characteristics. Filled and connected dots below each bar indicate which characteristics are included in that group of studies (e.g., a single dot indicates one characteristic only, whereas connected dots indicate studies including multiple characteristics). (**B**) Total number of studies conducted in younger, older, or mixed-age populations. (**C**) Proportion of studies within each exercise modality stratified by age group. (**D**) Age group distribution across intervention durations. (**E**) Total number of studies conducted in healthy, diseased, both, or unspecified populations. (**F**) Proportion of studies by health status across training modalities. (**G**) Proportion of studies by health status across training modalities. (**H**) Total number of studies conducted in sedentary, recreationally active (R.A.), trained, untrained, or unspecified populations. (**I**) Proportion of participants’ physical activity levels across different training modalities. (**J**) Proportion of physical activity levels across study durations.

Figure 6A presents an upset plot of participant characteristic combinations (i.e., intersections) identified across studies included in this review. Most studies included young (18–50 years, n = 464, 75.2%), old (≥51 years, n = 248, 40.2%), and diseased participants (n = 199, 32.3%). The most common intersection of participant characteristics involved the recruitment of young and healthy adults (n = 239, 38.7%; ). The intersections of older adults with comorbidities (n = 163, 26.4%), sedentary young adults (n = 129, 20.9%), and young adults with comorbidities (n = 98, 15.9%) were common in the included studies.

A total of 614 studies reported information regarding participant age. As seen in Figure 6B, most studies recruited only younger adults between 18 and 50 years old (n = 366, 59.6%). A total of 150 studies (24.4%) recruited only older adults (>50 years old), and 98 (16.0%) studies included both younger and older adults. Across aerobic training modalities (Figure 6C), most studies recruited younger adults. However, studies that recruited older adults generally examined longer training durations (Figure 6D).

As seen in Figure 6E, 259 studies included healthy participants (42%), while 184 recruited participants with defined health conditions (diseased [see Supplementary Document S4 for detailed definitions of diseases included]; 29.8%), or both healthy and diseased participants (n = 15; 2.4%). Of the 617 studies, 159 (25.8%) did not explicitly define the health status of their participants. Health status of participants across aerobic training modalities is shown in Figure 6F. Studies that recruited participants with disease were more commonly prescribed longer training durations (Figure 6G).

As seen in Figure 6H, most studies did not provide information regarding training status prior to enrolment (n = 267; 43.3%). Among the 350 remaining studies, sedentary (n = 161; 24.2%) and recreationally active (n = 105; 15.8%) individuals were most common. Figure 6I,J present baseline training status across aerobic exercise modalities and training durations. Interval protocols were used more in active populations (Figure 6I), while longer duration training interventions were more common among sedentary individuals (Figure 6J).

### 3.6. Risk of Bias Assessment

The risk of bias assessment for the subset of 65 studies evaluated is presented in the Supplementary Document S5. Overall, most studies had some concerns about the risk of bias. Forty-seven studies (72%) had at least one domain that was deemed to be of some concern for risk of bias, while 17 studies (26%) had at least one domain with high risk of bias. Only one study (2%) had a low overall risk of bias across all domains.

## 4. Discussion

This scoping review offers the most comprehensive mapping to date of the literature investigating the impact of aerobic exercise training on VO_2max_. Although publication frequency varied by country, the included studies originated from diverse geographic regions, highlighting widespread international engagement in VO_2max_ and aerobic training research. Our four major findings were: (1) a predominance of MICT studies compared with interval-based interventions, (2) a persistent underrepresentation of female participants, along with unbalanced sex distributions in many mixed-sex studies, (3) limited use of long-term training durations (e.g., >3 months), particularly in HIIT and SIT studies, and (4) inconsistent reporting of participant health and fitness characteristics coupled with overall poor reporting of disease and training statuses. While the direction of these trends may not be unexpected, no prior work has quantified their magnitude and persistence across 617 studies spanning 45 years.

### 4.1. Disparities in Aerobic Training Modalities

MICT is much more frequently studied than both HIIT and SIT, dominating the experimental work from the late 1900s captured in this review. Few HIIT or SIT studies appear before 2000 (Figure 3B), and the majority of studies still focused on MICT until the late-2010s (Figure 3C). This historical emphasis on MICT coincides with early physical activity guidelines [22,23,24,25], which prioritized moderate intensity exercise [26,27,28] on the principle that any form of physical activity was better than none [28,29]. Since the late 2010s, the proportion of studies that included a HIIT or SIT group has grown substantially (Figure 3C). These studies have found HIIT and SIT to be safe, feasible, and effective alternatives to MICT for improving VO_2max_ across a wide range of populations [30,31,32,33]. As evidence on the efficacy of HIIT and SIT for improving VO_2max_ accumulates, studies examining the optimal intensity of exercise training across all populations will continue to be needed. Importantly, the present scoping review describes trends in study design and representation and does not assess the comparative efficacy of aerobic training modalities. At present, the relative advantages of MICT, HIIT, and SIT on VO_2max_ remain contested, with some studies reporting larger improvements in VO_2max_ following higher intensity training [34,35,36], and others reporting no meaningful difference when total work is matched [37,38]. If evidence supporting the efficacy of HIIT and SIT for improving VO_2max_ continues to grow, these alternative training modalities should be incorporated alongside MICT in physical activity guidelines. Future studies that standardize training load, extend training duration, and emphasize diversity and inclusion will be essential to resolving this debate on the comparative effectiveness of aerobic training modalities.

### 4.2. Underrepresentation of Females in Exercise Science

A key finding of this review is the under-enrolment of female participants in aerobic training studies assessing VO_2max_ (Figure 4B). Males constituted most participants in every decade, and the cumulative female sample was markedly smaller (65% male vs. 35% female). This imbalance persists within individual studies, with female-only trials being rarer than male-only trials (Figure 4E). Importantly, this review is among the first to quantify sex imbalance within mixed-sex aerobic training studies, revealing that the inclusion of females frequently remains substantially lower than that of males (only 7.5% of 239 mixed-sex studies achieving parity (n = 18); more than half (n = 141; 59%) enrolled more males than females). Unequal sex ratios have important methodological implications as disproportionate group sizes reduce statistical power for detecting sex-by-training interactions, increase uncertainty around subgroup estimates, and can bias pooled effect estimates toward the more highly represented sex [39]. However, equal numerical representation alone does not guarantee higher-quality inference—especially when answering questions related to sex differences. Rather, the validity of sex-specific conclusions depends on intentional study design choices (including consideration of sex-specific biological variability), appropriate powering for interaction effects, and transparent reporting of sex-disaggregated outcomes. Future aerobic training studies should therefore justify sex composition a priori and ensure that sample sizes are sufficient to support the specific sex-based questions being asked.

Several factors likely fuel this gap. Historical concerns about hormonal variability, outdated views on female participation in sport, and tacit assumptions that training adaptations to exercise are uniform across sexes have discouraged female representation in the literature [40,41,42,43]. Authorship patterns may also play a role, as studies led by male first or senior authors enrol fewer female participants on average than those led by females, which underscores how a lack of female leadership in exercise science can perpetuate sampling biases [43]. Encouragingly, our temporal analysis (Figure 4) hints at gradual improvement with more recently (2020–2023) published studies having a higher proportion of female participants and a slight uptick in female-only study designs (Figure 4B,E). These shifts in recruitment patterns coincide with funding and regulatory agency mandates [40,43] for sex balance and a growing interest in sex-specific exercise responses. Future studies should design trials with balanced sex representation and power calculations that explicitly test sex-by-training interactions in an effort to develop training programmes that optimize VO_2max_ in females and males.

### 4.3. Limited Long-Term Aerobic Training Studies—Especially with HIIT and SIT

The duration of aerobic training interventions assessing VO_2max_ varied widely (Figure 5), with the overwhelming majority being shorter than three months. Interventions longer than six months almost always included MICT, of which, only 9 studies had training durations at least 1-year long (Figure 5). Interestingly, we did not observe a large increase in long-term interval-based studies during the past decade (2019–2023) despite growing research interest in HIIT and SIT programmes seen during this period. Given that improvements in VO_2max_, performance, and health continue to develop over months of training [44,45], it is unlikely that we currently understand the full adaptive potential of HIIT or SIT. At the same time, short-term interventions may also overestimate early gains in VO_2max_ as rapid initial improvements often attenuate with continued training [46,47], thereby leaving the long-term sustainability of VO_2max_ improvements following HIIT and SIT unclear [48]. Moreover, short intervention durations limit insight into adherence, tolerability, and long-term safety—factors that are critical for informing exercise prescription and guideline development across diverse populations. Until rigorous studies extend training interventions for HIIT and SIT beyond the typical 8–12 week window, the long-term efficacy and feasibility of these interval-based approaches will remain largely uncharacterized [48].

### 4.4. Limited Participant Characteristics and Experimental Reporting

Participant characteristics varied considerably across studies, and many studies failed to adequately report participant demographic information. Health and physical activity status were the most frequently underreported variables (Figure 6E,H). Emerging evidence indicates that health status is an important predictor of VO_2max_ response to aerobic training [49], with comorbidities such as ischemic stroke and diabetes associated with blunted improvements [50] and nonresponse [51]. Similarly, there is some evidence that the VO_2max_ response to aerobic training is inversely associated with baseline fitness status [52], with greater improvements in VO_2max_ observed among sedentary compared to trained individuals [53]. Without clear documentation of baseline health and fitness, it becomes difficult to distinguish true training effects from outcomes driven by pre-existing differences [54]. Therefore, future studies must adopt more consistent and transparent reporting of participant characteristics as they represent important confounders of training-induced changes in VO_2max_.

While formal risk-of-bias assessment is not required for scoping reviews [55], we included this analysis to provide illustrative context regarding common methodological considerations in the aerobic training literature. Our risk of bias assessment revealed that reporting practices of experimental methods were overall poor in a subset of our included studies (see Supplementary Document S5). These findings appear to coincide with our recent observations [56,57], and may suggest an ongoing need for greater methodological rigour and transparency in reporting across the exercise science literature.

### 4.5. Limitations

While this scoping review aimed to comprehensively capture the aerobic training literature assessing VO_2max_, we acknowledge several important limitations. Importantly, the total body of aerobic training literature is likely larger than what we captured (see Figure 1). We excluded studies that did not meet our criteria for appropriate VO_2max_ reporting to ensure consistency with our planned meta-analysis. Likewise, we also restricted inclusion to studies that prescribed aerobic training based on VO_2max_, VO_2peak_, or maximal workload (WR_max_). As a result, this review primarily reflects controlled, laboratory-based training studies and may underrepresent pragmatic or clinically oriented interventions that use alternative prescription methods (e.g., heart rate, rate of perceived exertion) or less intensive supervision models. However, exercise prescriptions based on VO_2max_, VO_2peak_, or maximal workload are widely used in aerobic training studies due to their validity and reliability for standardizing exercise intensity across participants [58]. Given the substantial number of studies included (n = 617), we believe this review still faithfully captures key trends, strengths, and limitations within the existing aerobic training literature and provides a solid foundation for future research.

## 5. Conclusions

To our knowledge, this scoping review is the first to comprehensively map the experimental literature investigating the effects of aerobic training on VO_2max_. By including 617 studies, we uncovered four gaps in the aerobic training literature that had remained largely implicit despite nearly five decades of research. First, there were far more studies using MICT than HIIT or SIT. Second, females remained under-represented and, when included, were often enrolled in smaller numbers than males. Third, less than one in ten studies extend beyond six months and almost none of these longer studies employ HIIT or SIT. Finally, reporting of participant health, baseline fitness, and key methodological safeguards was routinely incomplete. Future studies should target balanced male-to-female ratios, explicitly power sex-by-training interaction effects, and extend intervention durations beyond three months (particularly for HIIT and SIT protocols). Equally important is the adoption of transparent and rigorous reporting methods. Closing these gaps should strengthen our understanding of VO_2max_ adaptations following aerobic exercise, improve the external validity of training advice, and ultimately increase the clinical relevance of aerobic training interventions.

## Data Availability

The original contributions in this study are included in the article/Supplementary Material. Further inquires can be directed to the corresponding author. Registration of this project can be found on the Open Science Framework (osf.io/x9vu3).

## Supplementary Materials

The following supporting information can be downloaded at: https://www.mdpi.com/article/10.3390/jfmk11010070/s1. Supplementary Documents S1: Manuscript Information; Supplementary Documents S2: PRISMA Checklist; Supplementary Documents S3: Search Strategy; Supplementary Documents S4: Data Extraction Tool; Supplementary Documents S5: Risk of Bias; Supplementary Documents S6: VBA Methods; Supplementary Documents S7: Study Characteristics; Supplementary Documents S8: Citation Analysis; Supplementary Documents S9: Country of Origin.
